# Blackcurrant Alleviates Dextran Sulfate Sodium (DSS)-Induced Colitis in Mice

**DOI:** 10.3390/foods12051073

**Published:** 2023-03-02

**Authors:** Hye-Jung Moon, Youn-Soo Cha, Kyung-Ah Kim

**Affiliations:** 1 Department of Food Science and Human Nutrition, Jeonbuk National University, Jeonju 54896, Republic of Korea; 2 K-Food Research Center, Jeonbuk National University, Jeonju 54896, Republic of Korea; 3 Department of Food and Nutrition, Chungnam National University, Daejeon 34134, Republic of Korea

**Keywords:** blackcurrant, whole foods, ulcerative colitis, anti-inflammation, tight junction proteins, gut microbiota

## Abstract

Previous studies have reported that anthocyanin (ACN)-rich materials have beneficial effects on ulcerative colitis (UC). Blackcurrant (BC) has been known as one of the foods rich in ACN, while studies demonstrating its effect on UC are rare. This study attempted to investigate the protective effects of whole BC in mice with colitis using dextran sulfate sodium (DSS). Mice were orally given whole BC powder at a dose of 150 mg daily for four weeks, and colitis was induced by drinking 3% DSS for six days. Whole BC relieved symptoms of colitis and pathological changes in the colon. The overproduction of pro-inflammatory cytokines such as IL-1β, TNF-α, and IL-6 in serum and colon tissues was also reduced by whole BC. In addition, whole BC significantly lowered the levels of mRNA and protein of downstream targets in the NF-κB signaling pathway. Furthermore, BC administration increased the expression of genes related to barrier function: ZO-1, occludin, and mucin. Moreover, the whole BC modulated the relative abundance of gut microbiota altered with DSS. Therefore, the whole BC has demonstrated the potential to prevent colitis through attenuation of the inflammatory response and regulation of the gut microbial composition.

## 1. Introduction

Inflammatory bowel disease (IBD) refers to a chronic inflammatory condition of the intestinal tract that increases health and economic burdens due to an increase in global prevalence and lowers the quality of life [1]. Ulcerative colitis (UC), one of the typical IBDs, appears only in the colon and is marked by supercritical mucosal inflammation [2]. A cross-sectional study of 15 countries in Asia and the Middle East reported that UC is twice as prevalent as Crohn’s disease and occurs more frequently in men in their 30s [3]. In addition, it is essential to treat UC because it can develop into colorectal cancer if it persists for a long time [4]. UC is characterized by diarrhea, bloody stools, urgency, increased frequency of defecation, and, in severe cases, fever and weight loss [5]. It is estimated that UC is caused by the disruption of intestinal homeostasis due to genetic, microbiological, immunological, and environmental factors including diet, smoking, and stress [5,6]. Drugs such as 5-aminosalicylic acid (5-ASA), biological drugs (anti-tumor necrosis factor-α (anti-TNF-α) and anti-adhesion molecule inhibitors), immunosuppressants, and corticosteroids have been used to treat UC [5]. However, it has been reported that the remission rate of UC is only 15% to 44.9%, and adverse events such as infection, UC flare, nasopharyngitis, myelosuppression, liver toxicity, and malignancy occur [5,6,7,8]. Therefore, to develop other safe and effective treatments, natural products using polyphenols such as apigenin and curcumin, and polysaccharides such as *Scutellaria baicalensis* Georgi, are being studied [9,10].

Anthocyanins (ACN), belonging to the flavonoid subgroup of polyphenols, are found in flowers, vegetables, and fruits and are water-soluble pigments in red, blue, and purple [11]. Various health benefits of ACNs have been discovered, in particular, ACN supplements have been shown to improve gut health by modifying the gut microflora and enhancing the intestinal barrier, thereby reducing the potential risk of inflammation [11,12,13]. ACN-rich foods include berries (blackcurrants, blueberries, and raspberries) and dark red vegetables (red cabbage, eggplant, and purple wheat), among which blackcurrants have been reported to have a higher total ACN content than blueberries [11,14].

Blackcurrant (BC) has been suggested to possess various health effects, including prevention of obesity, improvement of cognitive impairment due to aging, and reduction of diabetes-related cardiovascular dysfunction [15,16,17]. Recently, ACN dietary supplements consisting of BC and bilberry extracts have shown anti-inflammatory effects in intestinal epithelial cells [18]. Additionally, silver nanoparticles based on BC extracts were observed to restore inflammation of induced colitis in mice [19]. However, these studies are insufficient to confirm the effect of BC on improving intestinal inflammation. Furthermore, most of these studies have verified the physiological activity of BC extracts, and studies on BC in its whole form are rare. Therefore, the aim of this study was to investigate whether the intake of whole BC in mice alleviates dextran sulfate sodium (DSS)-induced colitis.

## 2. Materials and Methods

### 2.1. Materials and Reagents

The commercial freeze-dried powder of whole BC was obtained from Sujon Berries (Nelson, New Zealand). According to Willems et al. (2017), 1 g of Sujon’s BC powder contained 23.1 mg of anthocyanin, 0.9 g of carbohydrates, and 8.2 mg of vitamin C [20]. DSS was bought from MP Biochemical (MW: 36–50 kDa; Solon, OH, USA). A TNF-α enzyme-linked immunosorbent assay (ELISA) kit was bought from Invitrogen (Vienna, Austria), and interleukin (IL)-1β and IL-6 ELISA kits were purchased from R&D Systems (Minneapolis, MN, USA). The RNAiso Plus kit, PrimeScript RT Master Mix, and bicinchoninic acid (BCA) protein assay kits were purchased from Takara Bio, Inc. (Shiga, Japan). RIPA buffer was procured from Thermo Scientific Inc. (Rockford, IL, USA). Primary antibodies including phosphorylated-p65 (p-p65), p65, inducible nitric oxide synthase (iNOS), cyclooxygenase-2 (COX-2), and β-actin were purchased from Cell Signaling Technology (Danvers, MA, USA).

### 2.2. Animals

The animal experiment was approved by the Animal Ethics Committee of Chungnam National University (IACUC approval number: 202112-CNU-214). Five-week-old male C57BL/6J mice were acquired from Central Lab Animal, Inc. (Seoul, Republic of Korea). The experimental design is illustrated in Figure S1. The mice were housed under the same conditions (temperature of 22 ± 2 °C, relative humidity of 50 ± 5%, and 12 h/12 h light/dark cycles) and acclimatized for six days. After the adaptation period, 24 mice were separated into three groups (*n* = 8 per group): Vehicle group, normal control group not treated with DSS; DSS group, DSS-treated control group; DSS + BC group, DSS and blackcurrant treatment group. In the DSS + BC group, BC powder diluted in phosphate-buffered saline (PBS) was orally administered at a dose of 150 mg/mice per day throughout the experimental period. The PBS dosage given to the Vehicle and DSS groups was the same as that given to the DSS + BC group. To induce colitis in the DSS group and the DSS + BC groups, 3% DSS (*w*/*v*) in drinking water was given for six days from the 21st day of the experiment. One day before the experiment’s termination, DSS was replaced with normal water.

Symptoms of colitis were monitored daily using the DAI (disease activity index) while DSS was administered. The DAI, which was slightly modified from what Peng et al. (2019) described, was measured as scores for body weight loss (0, none; 1, 1–5%; 2, 5–10%; 3, >10%), stool consistency (0, normal; 1, slightly loose feces; 2, loose feces; 3, watery diarrhea), and bloody stools (0, none; 1, slightly bloody; 2, bloody; 3, gross bleeding) [21]. Feces were collected the day before the sacrifice. Mice were euthanized after fasting for 12 h. The blood and colon tissues were obtained after the experiment was completed. Blood was centrifuged at 1100 g for 15 min to obtain the serum. After measuring the length and weight of colonic tissue samples, some were fixed in 4% formalin for histological assessment. The remaining colon tissues were immediately frozen in liquid nitrogen and kept at −80 °C until the experiment.

### 2.3. Histologic Analysis

Hematoxylin and eosin (H&E) staining was accomplished on 4 μm thick sections of colon tissues fixed in 4% formalin. Colon slides were examined using a light microscope (DM2500, Leica Microsystems, Wetzlar, Germany) installed at the Center for University-wide Research Facilities (CURF) at Jeonbuk National University (Jeonju, Republic of Korea). Histological damage to the colon tissue was evaluated by the scores of epithelium loss (0–3), crypt damage (0–3), depletion of goblet cells (0–3), and infiltration of inflammatory cells (0–3) [22].

### 2.4. Measurement of Inflammatory Cytokine Levels

Colon tissue was homogenized with lysis buffer, and the supernatant was separated. ELISA kits were used to quantify inflammatory cytokines (TNF-α, IL-1β, and IL-6) contained in the separated supernatant and serum, according to the manufacturer’s procedure.

### 2.5. Quantitative Real-Time PCR (qRT-PCR) Analysis

qRT-PCR analysis was performed with reference to Song et al. (2021) and the instructions of the manufacturer of the reagent [15]. Following the manufacturer’s directions for the RNAiso Plus kit (Takara Bio, Inc.), total RNA was extracted from the colon tissue. cDNA was synthesized from total RNA using PrimeScript RT Master Mix (Takara Bio, Inc.). The TOPrealTM SYBR green qPCR Premix (Enzynomics, Daejeon, Republic of Korea) and a 7500 real-time PCR system (Applied Biosystems, Foster City, CA, USA) were used to carry out the qRT-PCR. The relative expression of the target gene was determined using the 2 −ΔΔCt method and normalized to that of the internal reference GAPDH.

### 2.6. Western Blotting

Western blotting was carried out by referring to the experimental method of Jang et al. (2019) [22]. Total protein lysates were extracted by homogenizing the colon tissue in a radioimmunoprecipitation assay (RIPA) buffer containing protease and phosphatase inhibitors. The protein content of the supernatant obtained by centrifugation of the extract was quantified using a BCA assay kit. Loading buffer was added to the supernatant and inactivated at 95 °C for 10 min. Protein samples were electrophoresed on SDS–polyacrylamide gels and then transferred to polyvinylidene difluoride (PVDF) membranes. After blocking the membrane with 5% skim milk, the antibody diluted to an appropriate concentration was applied for 24 h at 4 °C. After washing the membrane with tris-buffered saline with 0.1% tween 20 (TBST), the secondary antibody was added, and the protein was identified using enhanced chemiluminescence (ECL) solution and the ChemiDoc system (ATTO LuminoGraph II, ATTO, Tokyo, Japan). The bands of the target proteins were quantified using Image J software (US National Institutes of Health, Bethesda, MD, USA) and normalized to β-actin.

### 2.7. Gut Microbial Community Analysis

Song et al. (2021) and Jang et al. (2019) were referred to for fecal collection and gut microbiota analysis [15,21]. The day after the DSS drinking was completed, feces were collected and stored at −80 °C in order to analyze the gut microbial community. The microbial community of the collected feces was analyzed by Macrogen Inc. (Seoul, Republic of Korea). In summary, a library for 16S metagenomic sequencing was prepared by amplifying the V3–V4 region of 16S rRNA using the Hercules kit on the Illumina platform to construct a library of DNA extracted from fecal samples. The sequencing results were analyzed using the QIIME2 program, and taxonomic information classification was confirmed using the BLAST program of the NCBI 16S database.

### 2.8. Statistical Analysis

Data were shown as the mean ± standard deviation (SD). Statistical analysis was performed using SPSS 18.0 software (SPSS Inc., Chicago, IL, USA). The significance of differences among groups was assessed using a one-way analysis of variance (ANOVA) by Duncan’s post hoc tests at *p* < 0.05.

## 3. Results

### 3.1. Effects of Blackcurrant on Clinical Symptoms and Colon Damage in DSS-Induced Colitis

**Figure 1:**
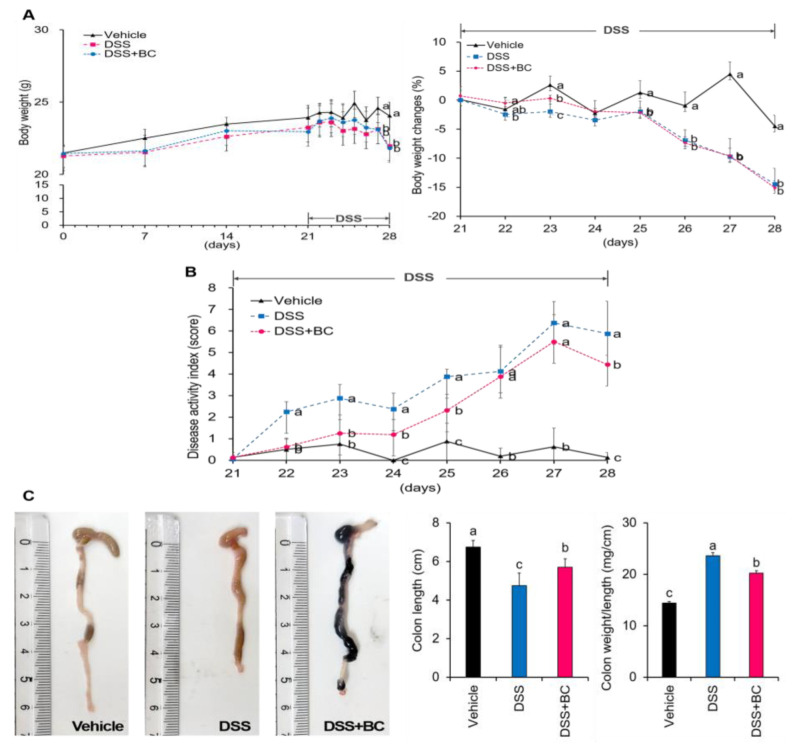
Effects of blackcurrant on the clinical symptoms of DSS-induced colitis. (**A**) Changes in body weight during the experiment period; (**B**) disease activity index (DAI); (**C**) representative images, and measurement of colon length and colon weight/length. The values are shown as mean ± SD (*n* = 8 per group). Distinct lowercase letters indicate significant differences between groups through one-way ANOVA and Duncan’s post hoc tests (*p* < 0.05). Vehicle, normal control group not treated with DSS; DSS, DSS control group; DSS + BC; DSS + blackcurrant group.

UC symptoms of colitis were identified as changes in body weight, disease activity index (DAI), colon length, and weight per length of the colon (Figure 1A–C). There was no significant difference in the change in body weight before DSS administration, but from the 6th day after DSS administration, both the DSS and DSS + BC groups were significantly reduced compared with the Vehicle control group (Figure 1A). Changes in DAI were checked daily during the DSS drinking period (Figure 1B). The DSS group showed a significantly higher DAI than the Vehicle group from the 22nd day. In contrast, the DSS + BC group showed significantly lower values than the DSS group until the 25th day. The DSS + BC group also showed an improved DAI on the final day of the experiment. The colon length was 4.73 ± 0.66 cm in the UC-induced DSS group, which was significantly shorter by about 29.9% compared with 6.75 ± 0.33 cm in the Vehicle group (Figure 1C). In the DSS + BC group, colon length was 5.70 ± 0.42 cm, and a DSS-related decrease in colon length was significantly restored. In addition, the DSS + BC group showed a significantly reduced colon weight-to-length ratio.

### 3.2. Effects of Blackcurrant on Histological Changes in the Colon Tissue in DSS-Induced Colitis

**Figure 2:**
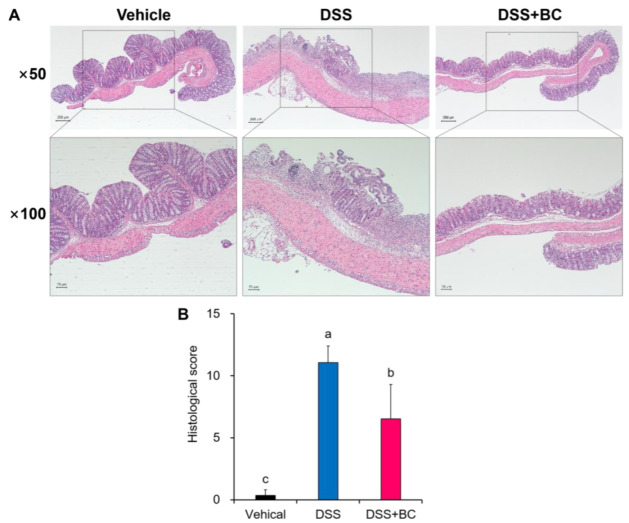
Effects of blackcurrant on the colon damage of DSS-induced colitis. (**A**) Representative images of microscopic colon tissue stained with hematoxylin and eosin (magnification 50× and 100×); (**B**) histology scores of each group. The values are shown as mean ± SD (five sections each within *n* = 3 per group). Distinct lowercase letters indicate significant differences between groups through one-way ANOVA and Duncan’s post hoc tests (*p* < 0.05). Vehicle, normal control group not treated with DSS; DSS, DSS control group; DSS + BC; DSS + blackcurrant group.

Sections of the colonic tissue were stained with H&E and histopathological scores were given to confirm the extent of damage (Figure 2A,B). The Vehicle group had no damage or inflammatory response to the mucosa, submucosa, crypt structure, or goblet cells in the colon. However, severe epithelial erosion, deficiency of goblet cells, destruction of the crypt structure, and infiltration of many inflammatory cells into the mucosa and submucosa were observed in DSS-treated mice. Supplementation with blackcurrant alleviated damage to the mucosal layer of colonic tissue and infiltration of inflammatory cells caused by DSS, and significantly reduced the histological damage score.

### 3.3. Effects of Blackcurrant on the Levels of Pro-inflammatory Cytokines in the Serum and Colon Tissue in DSS-Induced Colitis

The levels of proinflammatory cytokines in the serum and colon are shown in Table 1. The DSS group showed significantly higher levels of serum TNF-α and interleukin (IL)-6 than the Vehicle group. The DSS + BC group showed significantly attenuated levels of serum TNF-α, which were elevated by DSS. In colon tissue, the levels of TNF-α and IL-1β in the DSS group were increased significantly compared with the Vehicle group. However, the levels of TNF-α and IL-1β increased by DSS treatment were significantly reduced in the DSS + BC.

### 3.4. Effects of Blackcurrant on the Nuclear Factor-Kappa-Light-Chain-Enhancer of Activated B cells (NF-κB) Signaling Pathway, Tight Junction (TJ) Proteins, and Mucin in DSS-Induced Colitis

**Figure 3:**
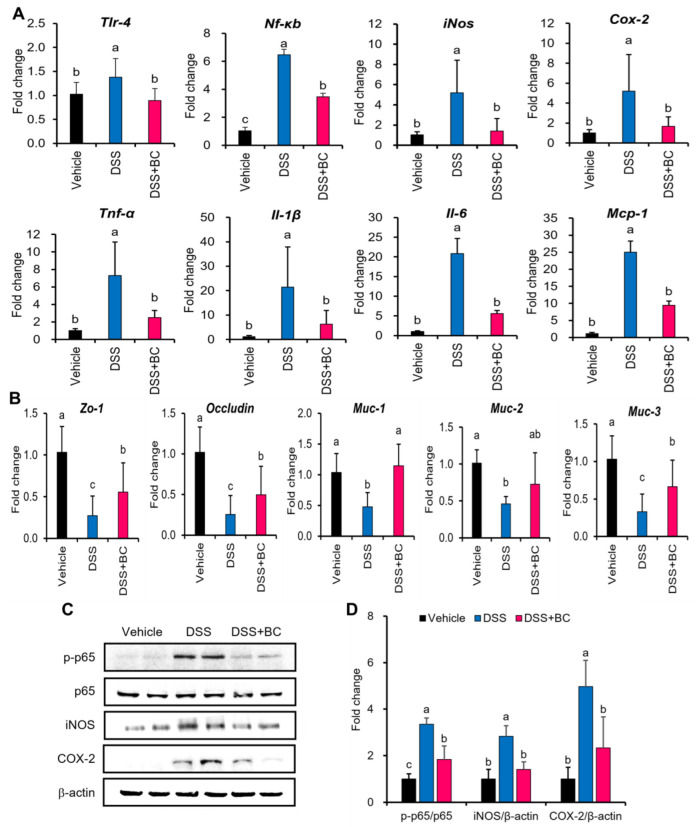
Effects of blackcurrant on the mRNA expression related to inflammatory factors, mucin, and tight junction protein, and the activation of the NF-κB signaling pathway in DSS-induced colitis. (**A**) The relative levels of mRNA expression related to inflammatory factors (*Tlr-4*, *Nf-κb*, *iNOS*, *Cox-2, Tnf-α*, *Il-1β*, *Il-6*, *Mcp-1*) in colon tissues; (**B**) the relative levels of mRNA expression related to tight junction protein (*Zo-1*, *Occludin*) and mucin (*Muc-1*, *Muc-2*, *Muc-3*) in colon tissues; (**C**) representative images and (**D**) quantitative results showing the expression levels of proteins (p-p65, p65, iNOS, COX-2) related to NF-κB signaling pathway in colon tissue. The values are shown as mean ± SD *(n* = 8 per group). Distinct lowercase letters indicate significant differences between groups through one-way ANOVA and Duncan’s post hoc tests (*p* < 0.05). Vehicle, normal control group not treated with DSS; DSS, DSS control group; DSS + BC; DSS + blackcurrant group.

We investigated whether BC affects the expression of genes and proteins related to the NF-κB signaling pathway, mucin, and TJ proteins (Figure 3A–D). The DSS group upregulated the genes of toll-like receptor-4 (TLR-4) and nuclear factor-kappa-light-chain-enhancer of activated B cells (NF-κB) related to the NF-kB signaling pathway compared with the Vehicle group (Figure 3A). Furthermore, an increase in the expression of iNOS, COX-2, pro-inflammatory cytokines (TNF-α, IL-1β, IL-6), and monocyte chemoattractant protein-1 (MCP-1), which are downstream genes of NF-κB, was observed in the DSS group. However, the expression levels of these excessive mRNAs were inhibited in the DSS + BC group, with a value similar to those of the Vehicle group.

Next, the effects of BC on the expression of genes encoding TJ proteins and mucin involved in barrier function were evaluated (Figure 3B). The DSS group significantly downregulated expression of all genes associated with TJ proteins and mucin compared with the Vehicle group. In contrast, the DSS + BC group showed higher expression of all such genes than the DSS group.

The expression of proteins related to the NF-κB signaling pathway, an inflammatory response pathway, was also examined (Figure 3C,D). As a result, it was found that the phosphorylation of NF-κB p65 (p-p65) and the protein expression of its downstream enzymes, iNOS and COX-2, were significantly increased in the DSS group compared with the Vehicle group. However, the DSS + BC group was revealed to inhibit the overexpression of p-p65, iNOS, and COX-2 increased by DSS. That is, it was shown that the administration of BC decreased the inflammatory response by inhibiting the NF-κB signaling pathway activated by DSS in the colon.

### 3.5. Effects of Blackcurrant on Modulation of the Gut Microbiome in DSS-Induced Colitis

**Figure 4:**
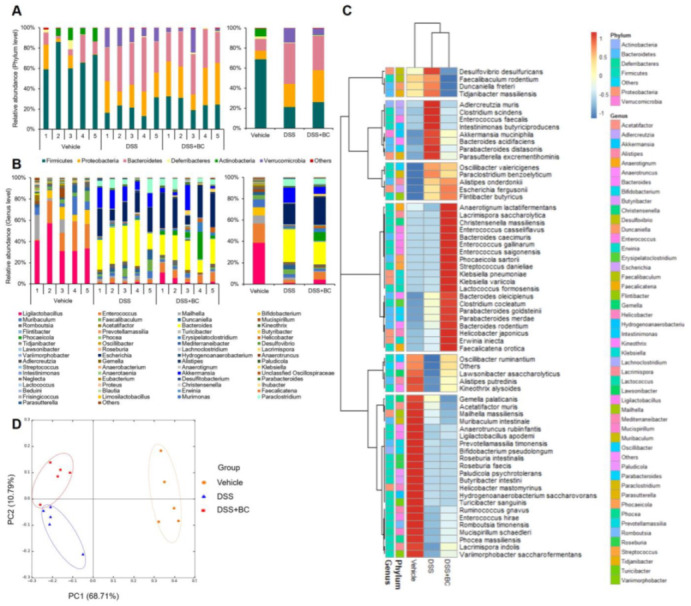
Effects of blackcurrant on gut microbiota on microbiota in DSS-induced colitis. (**A**) Taxonomy community analysis at the phylum levels; (**B**) taxonomy community analysis at the genus levels (≥0.03%); (**C**) heatmap analysis showing normalized abundance at the species levels; (**D**) principal coordinate analysis (PCoA) plots of gut microbiota of each group. The values are shown as mean ± SD (*n* = 5 per group). Vehicle, normal control group not treated with DSS; DSS, DSS control group; BC; DSS + blackcurrant group.

The influence of BC on the diversity and relative abundance of the gut microbiome was analyzed (Figure 4). To confirm the α-diversity of the gut microbiota, the observed amplicon sequence variant (ASV), an index of evenness, and Chao1, an index of richness, were evaluated. There was no significant difference between all groups, but the α-diversity of the DSS + BC group tended to increase slightly compared with the DSS group (ASV; Vehicle, 116.00 ± 32.33; DSS, 106.80 ± 9.36; DSS + BC, 125.20 ± 36.53, Chao1; Vehicle, 117.61 ± 32.30; DSS, 108.66 ± 11.33; DSS + BC, 127.79 ± 37.95).

Regarding the composition of gut microbiota, the DSS group showed a distinct alteration from that of the Vehicle group (Figure 4A–D). In taxonomic community analysis at the phylum level, *Firmicutes* and *Actinobacteria* were reduced in the DSS group compared with the Vehicle group, whereas *Bacteroidetes* and *Verrucomicrobia* were increased (Figure 4A). Meanwhile, the DSS + BC group was found to modulate the changes in the phylum caused by DSS. The abundance of *Ligilactobacillus*, *Enterococcus*, and *Bifidobacterium* at the genus level was high in the Vehicle group (Figure 4B). However, DSS treatment diminished these genera and elevated the levels of *Bacteroides*, *Escherichia*, and *Akkermansia*. BC decreased *Bacteroides* levels and increased *Ligilactobacillus* compared with the DSS group. Moreover, at the species level, the administration of BC was shown to regulate the change in microbial composition due to DSS (Figure 4C). As a result of analyzing β-diversity with a principal coordinate analysis (PCoA) plot to confirm the relative similarity in the gut microflora between each group, it was distinguished by the first principal component (PC1) between the Vehicle and DSS-treated groups (Figure 4D). Moreover, the DSS and DSS + BC groups were distinguished by the second principal component (PC2), and supplementation with BC tended to modulate the gut microbial community.

## 4. Discussion

The cause of colitis is considered to be an imbalance in intestinal homeostasis due to the influence of genetic, microbiological, immunological, and environmental factors [5,6]. Natural products are being developed to treat UC, and ACNs are known to have positive effects on gut health [9,10,12,13,18,19]. Thus, the current study aimed to analyze how the beneficial effects of ACN-rich BC caused immunological and microbiological changes in the colon in mice with DSS-induced colitis. Indeed, a previous study reported that nonalcoholic steatohepatitis was prevented in mice fed a high-fat/high-sucrose diet containing 6% whole BC powder, which was equivalent to consuming two cups of fresh BC per day in humans, for 24 weeks [23]. Based on a previous study, we explored the effect of oral administration of 150 mg/day (7.5 g/kg body weight (BW), total ACN content; 165 mg/kg BW) of whole BC powder to mice, which was less than the dose administered in the previous study. In addition, the anti-inflammatory effects in colitis mice induced by DSS when administered BC at this dose were confirmed as a result of this study.

Chemical induction of colitis using DSS in mice is the most widely used method because it reflects clinical symptoms and histological changes observed in humans [6,24,25]. DSS, which has a highly negative charge, acts directly on colonic epithelial cells as a chemical toxin and damages them, resulting in the depletion of mucin and goblet cells, epithelial erosion, and ulcers [24,25]. Destruction of the intestinal epithelial layer also increases colonic epithelial permeability, allowing commensal bacteria and related antigens to infiltrate the mucosa, followed by infiltration of immune cells such as neutrophils [22,24,25]. Immune cells infiltrating the lamina propria and submucosa reportedly secrete pro-inflammatory cytokines and disseminate inflammatory responses to underlying tissues [24,25]. The results of this work revealed that, when colitis was induced with DSS, clinical symptoms such as a decrease in body weight and colon length, as well as an increase in DAI and colon weight, were observed. Furthermore, histological changes were observed after inducing colitis with DSS, including epithelial loss, crypt damage, depletion of goblet cells, and infiltration of inflammatory cells. In contrast, the administration of BC had no effect on weight loss but showed beneficial effects on other clinical symptoms and histological changes following colitis induction.

In another study, the intake of 200 mg/kg BW of crude ACN isolated from the fruits of *Lycium ruthenicum* Murray had no effect on weight loss induced by DSS, similar to our results [21]. Previous studies also demonstrated that giving mice ACN-containing materials such as the water extract of maqui berry, ACN extracted from mulberry fruit and black rice relieved the pathological changes in the colon caused by DSS, like inflammatory cell infiltration and mucosal damage [26,27,28]. Additionally, when silver nanoparticles with a diameter of 213 nm based on blackcurrant extract were supplied to the DSS colitis mice model at a concentration of 2 mg/kg, only the macroscopic score and colon shortening were significantly improved [19]. Similar to the previous study, our study in which whole BC powder was administered also showed an improvement effect in these indicators, as well as a relieving effect in the colonic weight-to-length ratio. This difference is likely due to the difference in dose concentration.

Damage to intestinal epithelial cells caused by DSS was reported to worsen the inflammatory response by increasing the generation of pro-inflammatory cytokines [9,25,29]. It was also reported that the levels of TNF-α and IL-6 were altered in the serum of mice with early-stage colitis induced by one week of DSS administration [29]. Elevated levels of pro-inflammatory cytokines due to colitis can be reduced by various polyphenols, including ACNs [9,13,22]. In this study, except for IL-1β in the serum and IL-6 in the colon tissue, DSS treatment increased the levels of other pro-inflammatory cytokines, whereas BC administration decreased these levels. It was reported that treatment with petunidin 3-*O*-[rhamnopyranosyl]-(*trans*-*p*-coumaroyl)-5-*O*-[*β*-*D*-glucopyranoside] (P3G), isolated from the fruits of *Lycium ruthenicum* Murray, reduced all pro-inflammatory cytokines in the serum, but there was no difference in IL-1β levels in the crude ACN-administered group compared with the DSS-treated group, as in our study [21].

When mulberry ACN was administered, the inhibitory effect on pro-inflammatory cytokines in the colon decreased all indicators at a high concentration (200 mg/kg BW), but there was no change, except for IL-1β, at a low concentration (100 mg/kg BW) [26]. The major ACNs in BC are delphinidin-3-rutinoside, cyanidin-3-rutinoside, delphinidin-3-glucoside, and cyanidin-3-glucoside, and each food item contains different types of ACNs [11]. Therefore, the difference in effects on weight loss and pro-inflammatory cytokines was presumed to be due to differences in the types and intake of different ACNs in food, and differences in UC mouse models and disease stages. Moreover, previous studies have shown that BC extract decreases inflammation-related cytokines in bone-marrow-derived macrophages and vascular tissue in mice with type 2 diabetes mellitus [17,30]. Similarly, in the present study, BC was observed to reduce the production of pro-inflammatory cytokines, even when consumed in the form of whole BC powder.

Intestinal homeostasis is maintained by a barrier consisting of mucus, epithelial, and immune cells that prevent the penetration of bacteria and other antigens into the colon tissue [2,31]. DSS-induced loss of TJ proteins (ZO-1 and occludin) in mucus and mucin in the intestinal epithelial layers [21,26,31,32]. NF-κB is an inducible transcription factor that regulates the expression of genes encoding cytokines associated with immune and inflammatory responses and is involved in maintaining intestinal homeostasis [33]. When cells are stimulated externally through gut microbes, pro-inflammatory cytokines and toll-like receptors activate NF-κB (p-p65), which is known to be involved in the onset of inflammatory diseases by upregulating the expression of inflammation-related cytokines (TNF-α, IL-1β, and IL-6), chemokines (MCP-1), and inducible enzymes (COX-2, iNOS) [21,28,32,33].

In previous studies, the administration of ACN in mice with DSS-induced colitis and mice fed a high-fat diet increased the expression of factors related to mucin and TJ proteins in the colon, while downregulating the expression of target genes in the NF-κB signaling pathway [21,34]. In vitro, ACN-rich bilberry and BC extracts, as well as the 3-*O*-glucosides of cyanidin and delphinidin, have been shown to inhibit the activity of TNF-α-induced NF-κB in intestinal epithelial cells [18,35]. The results of this study demonstrated that BC intake enhanced the expression of genes related to mucin and TJ proteins in colitis-induced mice. Additionally, BC decreased the phosphorylation of the NF-κB subunit and downregulated the expression of NF-κB target genes and proteins, such as COX-2 and iNOS, which were shown to improve DSS-induced colitis.

Many studies have reported that changes in the community structure of gut microflora are associated with the development of colitis [10,21,22,24,25,26,28]. In the DSS-induced colitis model, maqui berry extract and ACNs of mulberry and *Lycium ruthenicum* Murray changed the α-diversity of gut microflora [21,28], but BC did not change it significantly.

However, it was confirmed that the treatment with BC had an effect on the β-diversity and gut microbial composition, which was distinct from that of the DSS group. Several studies using DSS-induced colitis mouse models revealed a reduction in *Firmicutes* and an increase in *Bacteroidetes* at the phylum level, and the intake of ACNs and flavonoids modulated their composition [36,37,38].

The genera *Lactobacillus* (some of the reclassified genera, *Ligilactobacillus* [39]) and *Bifidobacterium* in the colon, known to have beneficial effects on health in several studies, are reduced by DSS [22,28,40], and our results were similar. Similar to another chronic DSS animal study, this study observed that treatment with DSS increased the genus *Akkermansia*, and this increase was a positive correlation with IL-1β, a pro-inflammatory cytokine [40]. Although the genus *Akkermansia* is known to have anti-inflammatory effects, it is still controversial and more studies are required because its exact role in IBD is not known [41].

As a change in relative abundance at the species level, BC decreased *Bacteroides acidifaciens*, known colitis-associated bacteria, after DSS treatment, and increased *Bacteroides caecimuris*, which rose in the recovery phase after stopping DSS treatment [42]. In addition, BC administration tended to increase the abundance of *Mucispirillum schaedleri*, which has been reported to have a preventive effect against colitis caused by *Salmonella* and *Alistipes putredinis*, which decreases in IBD [43,44]. As such, BC modulated the composition of gut microbiota that was altered by DSS. However, further studies are required to investigate the precise mechanism for the role of gut microbiota in each in the alleviation of colitis by BC.

## 5. Conclusions

The intake of whole BC powder has been shown to prevent clinical symptoms and histological destruction caused by colitis. BC was observed to attenuate the levels of pro-inflammatory cytokines in serum and colon tissues and enhance the gene expression of mucin and tight junction proteins. Additionally, it downregulated the expression of target proteins and genes involved in the NF-κB signaling pathway. Furthermore, BC showed the potential to alleviate the intestinal inflammatory response by modulating the composition of gut microbiota altered by DSS. Therefore, in this study, whole BC powder showed a protective effect against DSS-induced colitis by regulating the inflammation-related NF-κB signaling pathway and gut microflora, confirming its potential as a natural dietary material to improve UC.

## Figures and Tables

**Table 1:** Effects of blackcurrant on the levels of pro-inflammatory cytokines in serum and colon tissues.

	Vehicle	DSS	DSS + BC
Serum (pg/mL)			
TNF-α	1.93 ± 0.58 ^c^	7.88 ± 1.28 ^a^	5.70 ± 1.07 ^b^
IL-1β	0.10 ± 0.00	0.12 ± 0.04	0.12 ± 0.04
IL-6	0.54 ± 0.05 ^b^	2.92 ± 1.13 ^a^	3.02 ± 0.51 ^a^
Colon tissue (pg/μg protein)			
TNF-α	28.75 ± 25.70 ^b^	65.05 ± 22.26^a^	37.30 ± 17.13 ^b^
IL-1β	2.85 ± 0.71 ^c^	37.58 ± 3.64 ^a^	25.30 ± 4.97 ^b^
IL-6	6.92 ± 0.57	7.12 ± 1.17	8.33 ± 1.45

The values are shown as mean ± SD (*n* = 8 per group). Distinct lowercase letters indicate significant differences between groups through one-way ANOVA and Duncan’s post hoc tests (*p* < 0.05). Values with no significant difference among the groups were indicated by blanks. Vehicle, normal control group not treated with DSS; DSS, DSS control group; DSS + BC; DSS + blackcurrant group.

## Data Availability

Data are contained within the article or Supplementary Materials.

## Supplementary Materials

The following supporting information can be downloaded at: https://www.mdpi.com/article/10.3390/foods12051073/s1, Figure S1: Experimental design.
