# Supplementary material for: Blackcurrant Alleviates Dextran Sulfate Sodium (DSS)-Induced Colitis in Mice
Source: Foods. 2023 Mar 2;12(5):1073. doi: 10.3390/foods12051073 (PMC10000425; doi:10.3390/foods12051073)
Supplement: Supplementary file 1 [file foods-12-01073-s001.zip › foods-2226735-supplementary.pdf]

Supplementary Materials

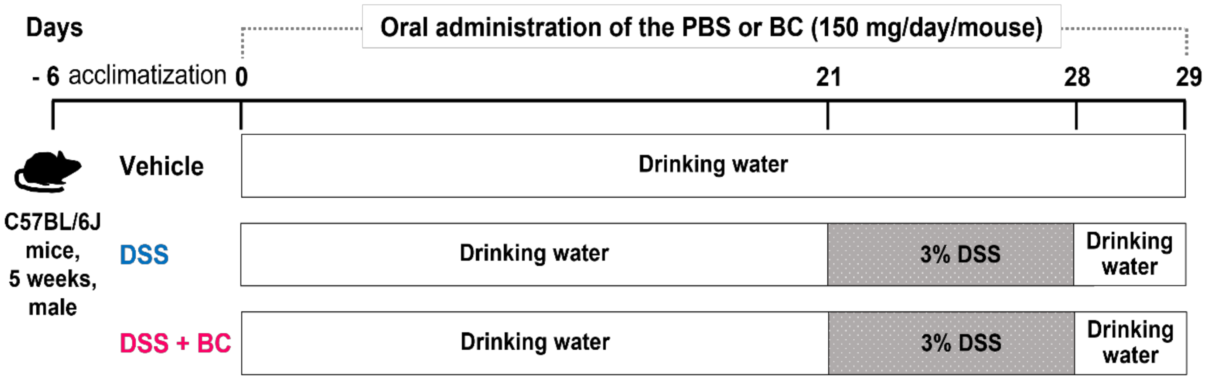

Figure S1. Experimental design. The experiment was performed for 29 days by dividing into the Vehicle group, DSS group, and DSS + Blackcurrant (BC) group. The sample was administered twice daily by oral administration to mice. The Vehicle group and the DSS group were given PBS whereas the DSS+BC group was given a freeze-dried Blackcurrant powder. After 21 days, colitis was induced for 6 days by 3% DSS in drinking water. Fecal was collected after DSS exposure.
